# The Sternocleidomastoid Muscle Reverse Pad: A “Safety Net” in Catastrophic Tracheal Surgery Situation

**DOI:** 10.3390/life14111423

**Published:** 2024-11-05

**Authors:** Sara Mantovani, Delia Giovanniello, Massimo O. Jaus

**Affiliations:** 1 Thoracic Surgery Department, San Camillo Forlanini Hospital, 00152 Rome, Italy; giovanniellodelia@gmail.com (D.G.); mjaus@scamilloforlanini.rm.it (M.O.J.); 2 Department of Cardio-Thoraco-Vascular Surgery, Sapienza University of Rome, P.le Aldo Moro, 00185 Rome, Italy; 3 Airway Surgery, Thoracic Surgery Department, San Camillo Forlanini Hospital, 00152 Rome, Italy

**Keywords:** tracheal surgery, tracheoesophageal fistula, airways surgery, sternocleidomastoid muscle

## Abstract

Background: This paper presents the outcomes of employing the inferiorly based rotated sternocleidomastoid muscle flap in complex tracheal reconstruction/repair scenarios, focusing on the key objectives of ensuring stable airway, functional digestive tract and patient survival. Methods: A retrospective analysis was performed for patients treated at two medical centers (A.O. San Camillo Forlanini, Rome, and A.O.U. Careggi, Florence) from 2011 to 2023, in which the sternocleidomastoid muscle (SCM) flap, detached from the mastoid and basicranium, was rotated on the lower pivot directly onto the repair site and pedicled to the sternal origin to ensure the continuity of the airway. Average postoperative hospital stay, follow-up period and patient survival were analyzed. Follow-up assessments encompassed bronchoscopies and CT scans conducted at intervals of 15 and 28 days, and subsequently at 3 and 9 months. Results: A total of five patients were enrolled in this study. These cases included one patient with anterior tracheal wall lesions with abundant tissue loss, one patient with an anterior wall necrosis due to descending cervical mediastinitis and three patients with extra-long tracheoesophageal fistulas (TEFs) (greater than 4.5 cm or >30% of the total tracheal length). In the case of the direct repair of a TEF with a proximal tracheal stenosis, the sternocleidomastoid muscle was used to reconstruct the tissue deficit caused by extensive loss of substance in the left lateral side of the tracheal wall. In case of repair through exclusion of the TEF, the sternocleidomastoid muscle was interposed between the visceral sutures after exclusion of the TEF by an endomechanical device, in one case even substituting the membranous part of the tracheal wall. Our technique allows rotation on the sternal head of the sternocleidomastoid muscle with the lowest rotation radius, pedicled to the sternal origin, detached from the mastoid process and superior nuchal line, thus providing optimal vascularization from the superior thyroid artery/external carotid artery and accessory vasculature from the suprascapular artery. Patients exhibited uneventful postoperative recovery concerning airway and digestive patency. The mean postoperative hospitalization duration was 41 days. The follow-up assessments were negative for postoperative complications. Conclusions: The use of sternocleidomastoid muscle flap was proposed to ensure repair and protection of the suture margin or to constitute a portion, as a scaffold, of the wall by leveraging the muscle’s vascularization and thickness. This technique may be considered a leading component in managing complex situations in tracheal surgery.

## 1. Introduction

Lesions of the upper airways are not common, but their management is complex, especially when the digestive tract is involved. Restoring the continuity of the airway and digestive tract when necessary is not always possible without the need of complex reconstructions, especially when there is a severe tissue loss. The literature contains many examples of maxillofacial reconstructive surgery using sternocleidomastoid muscle flaps to reconstruct tissue loss in oral cavity or pharyngeal surgery, or even for the closure of pharyngocutaneous fistulas [1]. In this paper, we aim to present our experience in using vascularized sternocleidomastoid flaps to restore the upper airway in complex situations involving severe tissue loss. Our technique involves the preparation and rotation of the sternal head of the SCM, preserving its vascularization, to cover the airway defect. The vascular anatomy of the SCM and the surgical technique will be thoroughly explained in the specific sections of this article.

## 2. Materials and Methods

We retrospectively collected data from five patients who were referred to us for complex airway lesions between 2011 and 2023. The medical history data of these patients are shown in Table 1. The study was conducted in accordance with the Declaration of Helsinki.

The inclusion criteria for the study were (1) tissue loss of the trachea exceeding 50% of its length or the presence of a tracheoesophageal fistula extending over 30% of the tracheal length, (2) complete hospitalization and follow-up data. The exclusion criteria were (1) incomplete clinical or follow-up documentation, (2) patients with airway lesions treated with direct repair or other techniques without use of SCM flap and (3) patients who have undergone previous neck surgery.

Informed consent was obtained from all subjects involved in the study.

Of these five patients, two presented airway lesions only. The first had an extensive lesion of the anterior wall of the trachea due to trauma from a chainsaw accident. The defect extended over 50% of the total length of the trachea. The second patient had necrosis of the anterior wall of the trachea due to descending mediastinitis; in this case as well, the defect extended over 50% of the total length of the trachea. In both cases, the tissue loss was in the proximal half of the trachea.

The remaining three patients had a tracheoesophageal fistula measuring over 4.5 cm or more than one-third of the total length of the trachea. In two cases, the fistula was secondary to the creation of a tracheostomy; in the last patient, the fistula developed after an Ivor Lewis esophagectomy.

All patients underwent follow-up with fiberoptic bronchoscopy and CT scans of the neck and chest at 15 and 28 days, and at 3 and 6 months after the surgery.

Statistical analysis was conducted using descriptive methods, given the small sample size of patients.

## 3. Results

The sternocleidomastoid muscle, due to its anatomy, has the potential to be pedicled and transposed in various ways [2]. In our technique, the criteria we considered necessary are (1) the muscle flap must be long enough to cover the defect without tension and (2) the muscle flap must maintain the best possible vascularization to prevent necrosis.

### 3.1. SCM Anatomy

The sternocleidomastoid muscle consists of two superficial heads, the clavicular and sternal heads, both of which insert into the mastoid process of the temporal bone. It also has a deep head extending from the inner third of the clavicle to the mastoid process. Its blood supply is divided into three main levels: (1) superior, originating from the occipital artery and its branches; (2) middle, supplied by the superior thyroid artery; and (3) inferior, fed by the suprascapular artery. In the first two cases, the blood supply comes directly from the external carotid artery, while in the third case, it comes from branches of the subclavian artery. This complex and interconnected vascularization makes the SCM an excellent muscle for being pedicled and used in reconstructive surgery.

### 3.2. Surgical Technique

**Figure 1:**
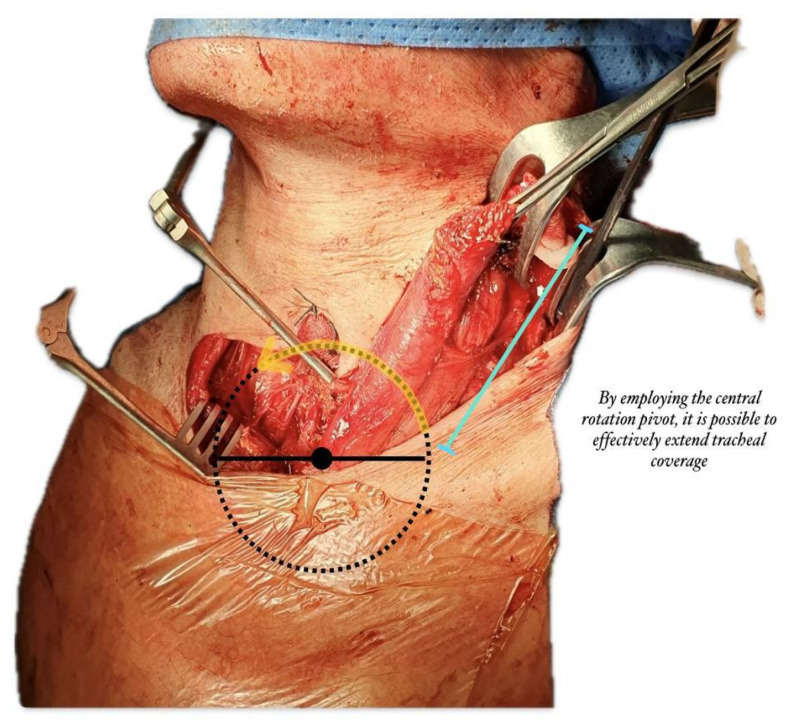
**Schematic view of SCM flap rotation:** by employing the central rotation pivot, it is possible to effectively extend tracheal coverage.

Our technique allows rotation on the sternal head of the sternocleidomastoid muscle with the lowest rotation radius, pedicled to the sternal origin and detached from the mastoid process and superior nuchal line, thus providing optimal vascularization from the superior thyroid artery or external carotid artery and accessory vasculature from the suprascapular artery (Figure 1).

In case of direct repair, the SCM flap was used to replace the loss tissue loss, restoring the physiological continuity of the airway. Therefore, in the first two patients, the sternal head of the sternocleidomastoid muscle was pedicled, maintaining its vascularization, and rotated medially by approximately 45 degrees. Then, it was used to support the reconstruction of the cartilaginous part of the trachea.

**Figure 2:**
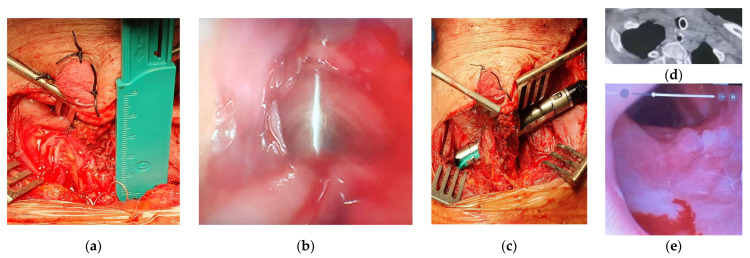
The patient presents a 4.5 cm tracheoesophageal fistula (**a**) due to tracheostomy performed for respiratory failure. Under endoscopic guidance (**b**), we perform TEF stapling (**c**) and we interpose SCM flap between the visceral sutures. One month after surgery, CT scan (**d**) and endoscopic control (**e**) are negative.

In one patient with a tracheoesophageal fistula clinically not suitable for repair by tracheal resection and anastomosis, the SCM flap was interposed between the two visceral sides of endomechanical sutures to protect them (Figure 2). In one young patient affected by a TEF and proximal tracheal stenosis clinically suitable for repair by tracheal resection and anastomosis and direct double layer suture of the esophageal defect, we used the SCM flap to cover a left-side lateral defect of the cartilaginous wall of the airway that could not be closed by a tracheo-tracheal anastomosis. Additionally, in one of these patients, the SCM flap allowed the reconstruction of the missing membranous part over a long segment.

All patients enrolled in the study completed the follow-up with CT scans and fibrobronchoscopy at 15 and 28 days after surgery, and at 3 and 9 months. No patient experienced complications of the airway or the upper digestive tract, either in the short term or the long term. The general information regarding surgery and follow-up is presented in Table 1; a serial number was assigned to each case.

## 4. Discussion

The use of SCM flaps in reconstructive surgery of the neck and head has been known for over half a century It was first described in 1955 by Owens [3] for the reconstruction of the oral cavity, and until the 1980s, this type of flap was used exclusively for maxillofacial reconstructive surgery. The anatomy and unique vascularization of the SCM make it a particularly versatile muscle, capable of reaching the structures of the face, oral cavity and neck. After Owens, other authors reported various facial reconstructive techniques using the sternal or clavicular head of the SCM, such as Schottstaedt [4] in 1955 and Dingman [5] in 1969. The use of pedicled SCM flaps is also described in patients with various types of neck tumors, where local treatments (surgical and/or radiotherapeutic) make the dissection and preservation of proper flap vascularization difficult. For this reason, studies report total and partial flap loss rates ranging from 10 to 30% [6]. Subsequently, cases of high esophageal fistula repairs using an SCM flap were described. In 2014, Nakajimaa et al. [7] presented their experience with the dehiscence of cervical esophagogastric sutures following McKeown procedures; to cover the fistula, they used a vascularized SCM flap with excellent results [8].

Reconstructive airway surgery is always tricky and challenging [9]. Tissue loss might not be restorable by direct suture, or the patient could not be clinically suitable for tracheal resection and reconstruction [10].

The sternocleidomastoid muscle has two main vascular supplies as explained in the text: a dominant one and a secondary one from the superior thyroid artery, which primarily supplies the sternal head of the SCM. In 2021, Srivastava et al. studied and demonstrated that preserving the vascularization from the superior thyroid artery, even in the absence of blood supply from the occipital artery branches, allowed the maintenance of an acceptable flap viability [11].

The surgical technique we present involves preserving the vascularization from the superior thyroid artery during the dissection of the sternal head of the SCM. Additionally, the rotation of the flap prepared in this manner has a smaller radius, creating less tension. This allows for the use of this robust and well-vascularized flap in airway reconstructions when there is significant tissue loss.

In the case of tracheoesophageal fistulas, the presence of the muscle flap, in addition to the previously described advantages, protects the visceral sutures by being interposed between them [12]. This results in stability of the airway and the replacement of long segment of membranous part. In their study, Alexandre Pl et al. described six patients in whom they used the infrahyoid muscle to close the tracheoesophageal fistula after laryngectomy; in their experience, they proposed this technique as a valid alternative to the SCM flap. However, this can only be applied if the infrahyoid muscle has been previously preserved and in the presence of small fistulas [13]. The technique we propose by interposition of the SCM flap allows us to address even extremely complex situations with significant tissue loss.

Lurin IA et al. presented the use of an SCM flap in a patient with a laryngopharyngeal injury from a firearm in the context of the war in Ukraine. After removing the metal fragments, the pharynx was repaired with direct suturing, while the destroyed thyroid cartilage was replaced with a SCM flap [14].

In conclusion, based on our experience, the use of a muscle flap composed by the sternal head of the SCM appears to be an excellent option in situations where restoring the integrity of the airway is complex or impossible due to severe tissue loss. This technique is highly effective, ensuring good vascularization of the flap, providing good airway stability and protecting the visceral sutures when intervention on the digestive tract is necessary.

## Figures and Tables

**Table 1:** Patient’s data.

	Sex	Age (y)	Cause	Diagnosis	Treatment	Tracheo	Hospital Stay (Days)
1	M	65	Iatrogenic (post Ivor Lewis esophagectomy)	Large TEF	Transtracheal fistulorrhaphy intertracheoesophagela apposition of SCM on the tracheal side	Yes	42
2	M	46	Descendant cervicomediastinitis (initial tonsillitis)	Anterior tracheal wall necrosis	Right SCM flap rotated to cover the substance loss	Yes	52
3	M	35	Iatrogenic (post-intubation)	Large TEF wit loss of substance of the left lateral tracheal wall	TEF repair, resection and ETE tracheal anastomosis, left SCM flap to cover the substance loss (including membranous part of trachea)	Yes	46
4	M	62	Chainsaw wound	Large lacerated contused wound at the anterior neck region + anterior portion of the sternum with extensive tracheal lesion with loss of substance	Reconstruction of the large tracheal laceration and left SCM flap rotated to cover the substance loss	Yes	34
5	M	72	Iatrogenic (post-trachetomy)	TEF	Exclusion of TEF with mechanical stapler and intertracheoesophageal interposition using left SCM flap	Yes	30

The table shows the clinical data of the patients included in the study. All patients are male, but this is entirely coincidental and not statistically significant due to the small sample size. The average total hospital stay is 40.5 days.

## Data Availability

Dataset available on request from the authors. The raw data supporting the conclusions of this article will be made available by the authors on request.
